# Role of Cell-to-Cell Variability in Activating a Positive Feedback Antiviral Response in Human Dendritic Cells

**DOI:** 10.1371/journal.pone.0016614

**Published:** 2011-02-08

**Authors:** Jianzhong Hu, German Nudelman, Yishai Shimoni, Madhu Kumar, Yaomei Ding, Carolina López, Fernand Hayot, James G. Wetmur, Stuart C. Sealfon

**Affiliations:** 1 Department of Microbiology, Mount Sinai School of Medicine, New York, New York, United States of America; 2 Department of Neurology, Mount Sinai School of Medicine, New York, New York, United States of America; 3 Center for Translational Systems Biology, Mount Sinai School of Medicine, New York, New York, United States of America; University of Texas Medical Branch, United States of America

## Abstract

In the first few hours following Newcastle disease viral infection of human monocyte-derived dendritic cells, the induction of *IFNB1* is extremely low and the secreted type I interferon response is below the limits of ELISA assay. However, many interferon-induced genes are activated at this time, for example *DDX58* (RIGI), which in response to viral RNA induces *IFNB1*. We investigated whether the early induction of IFNBI in only a small percentage of infected cells leads to low level IFN secretion that then induces IFN-responsive genes in all cells. We developed an agent-based mathematical model to explore the IFNBI and DDX58 temporal dynamics. Simulations showed that a small number of early responder cells provide a mechanism for efficient and controlled activation of the DDX58-IFNBI positive feedback loop. The model predicted distributions of single cell responses that were confirmed by single cell mRNA measurements. The results suggest that large cell-to-cell variation plays an important role in the early innate immune response, and that the variability is essential for the efficient activation of the *IFNB1* based feedback loop.

## Introduction

The innate immune response to viral infection is essential in fighting infections, and the dynamics of the response can determine whether the infection evolves into pathology [1]. Dendritic cells (DCs) are the primary response cells mediating the progression from innate to adaptive immunity and the induction of self-tolerance [2], [3]. It has recently been recognized that understanding the internal dynamics of DCs following viral infection can help elucidate the function of the immune system and help lead to better vaccination protocols [4], [5], [6], [7]. Many viruses have evolved immune antagonists that subvert the innate immune response and facilitate their replication. Newcastle disease virus (NDV), however, is an avian virus that lacks a functioning immune antagonist for human cells [7], [8], [9]. Therefore it efficiently stimulates the innate immune response and represents an ideal stimulus with which to probe the activation dynamics of human DCs.

Upon viral infection of DCs, constitutive RIG-I proteins (coded from the gene *DDX58*) can be activated upon detecting evidence of negative stranded viruses such as NDV in the cytoplasm, leading to *IFNB1* induction through a signaling cascade. *IFNB1* encodes IFNβ, which is a type 1 interferon (IFN) that is secreted into the extracellular medium, where it binds to IFN cell surface receptors on the secreting cell and on neighboring cells. This binding activates a gene program that plays an essential role in both the DC antiviral response [10] and in DC maturation [9], [11], and causes the up-regulation of many genes [12]. In particular, IFNβ binding activates the Jak/Stat pathway, inducing the *DDX58* gene and leads to RIG-I production. In infected cells, the activation of newly induced RIG-I, as well as of other genes involved in interferon induction such as IRF7 [13], leads to additional *IFNB1* production, thus completing an *IFNB1*-*DDX58* positive feedback loop.

Previous single cell measurements of *IFNB1* induction in NDV infected human monocyte-derived dendritic cells (MDDCs) showed large cell-to-cell variation ranging over 3–4 orders of magnitude at 8–10 hours after infection. These results, complemented by simultaneous measurements of *DDX58* (coding for RIG-I) production at 6 and 10 hours, are confirmed by the experiments presented here. Taking as a starting point the observed stochasticity of the cellular *IFNB1* response, we developed a stochastic agent-based model (ABM) that, after a fit to the later time data points, allowed extrapolation to early times after infection, where direct measurements are very difficult due to sampling limitations. Model simulations suggest that there is a small subset of early responder cells responsible for propagating cellular resistance to viral infection through the efficient activation of the *IFNB1-DDX58* feedback loop. The existence of such a subset resolves an apparent paradox of average cellular response, namely the fact that contrary to the temporal order of the feedback loop, *IFNB1* production remains near the limit of detection until 6 hours after infection, whereas that of *DDX58*, induced by IFNβ through the feedback loop, reaches a significant level of expression several hours earlier. This paradox in the average response illustrates how average cell responses can obscure the actual single cell response dynamics and support the role of cell-to-cell response variability in maintaining an efficient and controlled immune response.

## Results

**Figure 1:**
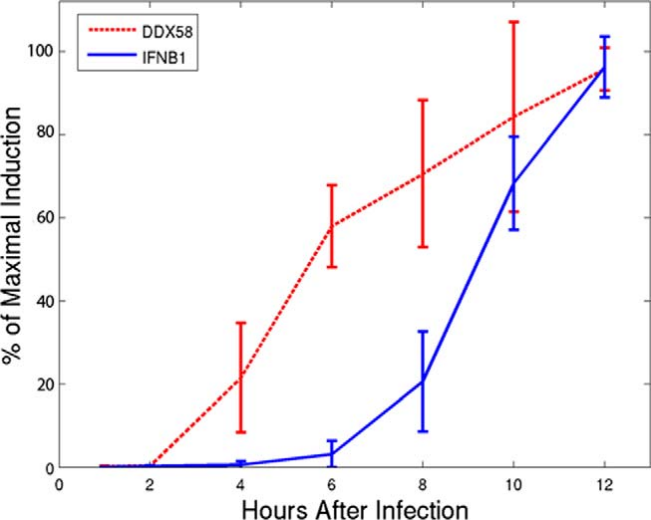
Time course of *IFNB1* and *DDX58* induction. Measurement of *IFNB1* (solid line) and *DDX58* (dashed line) expression in human DCs at 1, 2, 4, 6, 8, 10, 12, 14, 16, and 18 hours following NDV infection. Percent of maximal induction was measured by microarray, compared to non-infected control, and shows that half-maximal induction of *DDX58* occurs hours prior to *IFNB1* half-maximal induction.

The population (average) levels of *IFNB1* and *DDX58* transcripts in human MDDCs were measured as a function of time following NDV infection at multiplicity of infection, MOI = 0.5, as shown in **Figure 1** (see also Supplementary **Figure S1**). Our previous work [14] showed that at ten hours post infection both genes have reached approximately maximal expression levels. Half-maximal *DDX58* expression occurred approximately 3 hours after infection, while half-maximal *IFNB1* expression occurred 4–5 hours later. The time course for synthesis of the NDV L gene at 0, 3, 6, and 10 hours post infection (Supplementary **Figure S1C**) showed significant NDV genome replication well before the half-maximal expression level of *IFNB1* was reached. *MXA* induction (Supplementary **Figure S1D**) mimicked that of *DDX58* (Supplementary **Figure S**1B), and *IFNA1* induction (Supplementary **Figure S1E**) mimicked that of *IFNB1* (Supplementary **Figure S1A**), as expected from their common activation pathways. However, since *DDX58* is an interferon-induced gene, we would expect its expression to increase only after *IFNB1* expression increases.

Paradoxically, the reverse temporal order is observed (**Figure 1A** and Supplementary **Figure S1**). However, close inspection of the data (Supplementary **Figure S2A**) shows evidence for an initial and small first phase of *IFNB1* induction that begins as early as 1 hour after infection and never reaches levels above 1% of maximum until the sizeable induction observed after 6 hours. To evaluate the robustness of this low level *IFNB1* induction, we performed similar experiments with MOI = 0.1, 0.5, 1.0, 2.0 and 5.0 (Supplementary **Figure S2**), and obtained similar results. These findings demonstrate that an early low level of induction of *IFNB1* precedes - and therefore might be responsible for - the induction of *DDX58*.

**Figure 2:**
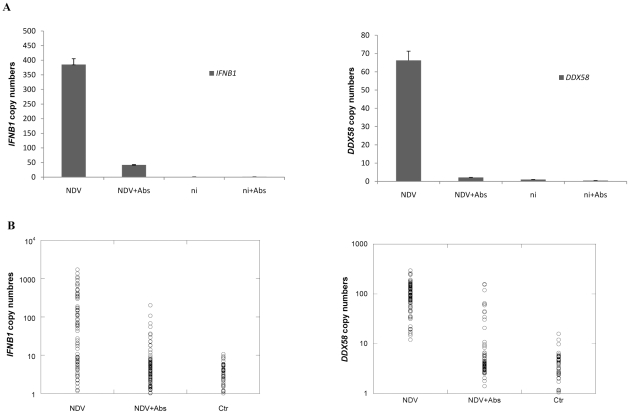
*DDX58* and *IFNB1* mRNA expression level. 2A. Total mRNA copy number. *DDX58* and *IFNB1* mRNA were measured by quantitative real-time PCR and the relative *IFNB1* and *DDX58* expression levels were normalized to *ACTB*. The columns show the gene expression level 7 hrs post treatment [NDV only (NDV), NDV plus blocking antibodies (NDV+Abs), not infected (ni), not infected with blocking antibodies (ni+Abs)]. Left panel: *IFNB1.* Right panel: *DDX58*. Error bars represent measurement error. 2B. Single cell mRNA expression. *DDX58* and *IFNB1* mRNA in individual DCs were measured 7 hrs post treatment by the hemi-nested PCR protocol illustrated in Supplementary **Figure S4**. The copy numbers of both *DDX58* and *IFNB1* in single DCs (normalized to *ACTB*) were determined for all cells with detectable expression of the mRNAs. Each symbol shows the gene expression in a single cell. The treatments were same as those in **Figure 2A**, but with uninfected DCs labeled as control (Ctr). Left panel: *IFNB1*. Right panel: *DDX58.*

The role of this early IFNβ in the induction of *DDX58* was tested by performing population and single cell assays measuring *IFNB1* and *DDX58* in the presence and absence of antibodies that attenuate extracellular interferon signaling. Cells were infected at an MOI = 0.5. As shown in **Figure 2A**, a mixture of antibodies binding interferons and the interferon receptor eliminated the induction of *DDX58* and dramatically reduced induction of *IFNB1*, confirming the formulation that IFNβ produced from the nearly undetectable levels of early *IFNB1* is responsible for the apparently paradoxical induction of *DDX58*.

As only half the cells were infected, in order to determine the distribution of responses observed in individual cells, we performed single cell mRNA assays (**Figure 2B**). Notably, in the absence of blocking antibodies about half the cells showed induction of *IFNB1,* while all cells showed induction of *DDX58.* In the presence of blocking antibodies, most cells showed control levels of both transcripts. Because *IFNB1* is an intronless gene, the control levels of expression reflect the 4 DNA strands encoding the gene within each cell (the sense and the anti-sense DNA strands of the gene on both alleles). In the presence of blocking antibodies, a few cells were detected that showed a modest induction of *INFB1* or of *DDX58* greater than the levels measured in control cells.

The results shown in **Figure 2B** showed significant cell-to-cell variability in the expression level of *IFNB1,* which ranged over three orders of magnitude. One factor that can differ among cells is their degree of differentiation, which is reflected in the level of the differentiation marker CD14. In order to test whether this variability contributes the large variation in gene induction with virus infection, we sorted cells into high and low CD14 subpopulations prior to infection with virus. In both groups, a similar and broad distribution of single-cell *IFNB1* gene responses was observed (Supplementary **Figure S3**). These results suggest that the heterogeneous levels of *IFNB1* in individual cells does not result from cell heterogeneity in differentiation and reflects both the absence of expression in uninfected cells and the noise in the transcriptional induction of this gene.

What is the source of the low level of *IFNB1* detected at early time points after virus infection that is necessary to initiate the positive feedback loop and generate a full antiviral response? Given the high intercellular variation in responses, the most parsimonious hypothesis is that a few infected cells are competent to induce *IFNB1* before interferon activation of JAK-STAT signaling. We explored this hypothesis and its implications by developing a formal model of the system that could lead to testable predictions.

The model was agent-based (ABM) stochastically simulating intercellular IFNβ signaling and the temporal evolution of the immune response in individual cells. The constitutive RIG-I distribution across cells, and the parameters of *IFNB1* induction were taken such that only the small number of cells with large amounts of RIG-I protein responded to infection. (see Materials **and Methods** **and** for additional details). The model can be accessed at http://tsb.mssm.edu/DCresponse2viralInfABM.

**Figure 3:**
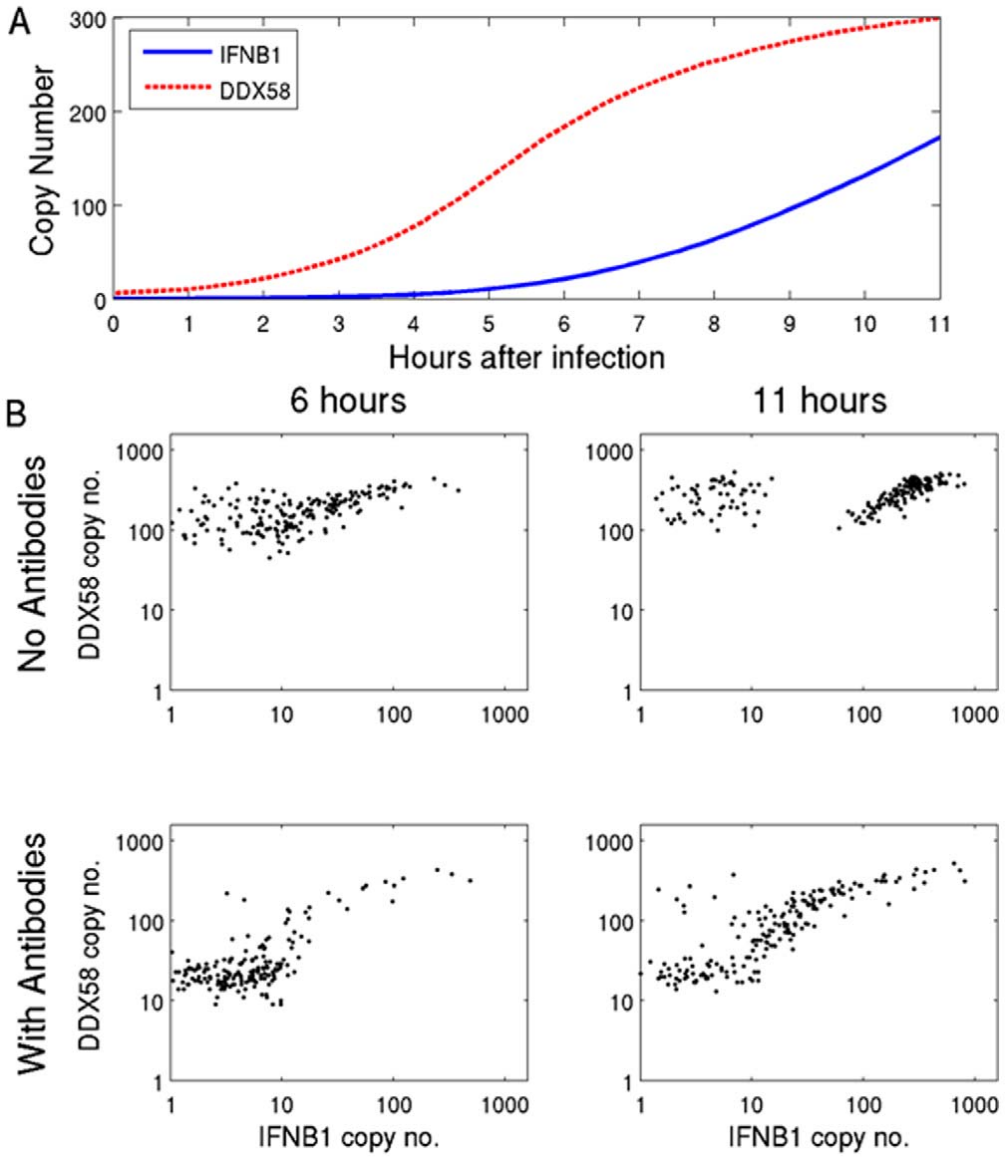
Single DC simulations with and without IFN-blocking antibodies. 3A. Time course of the average copy number of *IFNB1* (solid line) and of *DDX58* (dashed line) obtained from the simulation with the ABM model. 3B. Scatter plots display *IFNB1* (X axis) and *DDX58* (Y axis) mRNAs copy numbers in each single DC as simulated with the ABM model. Simulations without blocking antibodies (upper panel) or with blocking antibodies (lower panels) are shown at 6 hrs (left) and 11 hrs (right) post infection.

**Figure 3A** shows the time courses of the average induction of *IFNB1* and of *DDX58* obtained in the simulation, which are consonant with the experimental results shown in **Figure 1A**. A more stringent test of the model is provided by the distributions of single cell results obtained by simulation (**Figure 3B**). The model was simulated using a constant parameter set both with and without blocking antibodies and the pattern of responses in single cells was determined. The antibody blockage was implemented in the model by introducing a degradation rate for extracellular interferon β protein, which allowed it time to bind to the cell that secreted it, but made it unlikely that it would reach a neighboring cell. The effect of receptor antibody was included by reducing the receptor binding rate. Uninfected cells can be roughly characterized as those with less than ten copies of *IFNB1*. The RIG-I mRNA distribution in uninfected cells was considerably shifted downward when antibodies blocked the paracrine loop (**Figure 3B**), as the paracrine loop acts as an inducer of RIG-I. Furthermore, *DDX58* and *IFNB1* showed significant correlation within individual cells at 11 hours, as expected when each cell is activated only by autocrine signaling.

**Figure 4:**
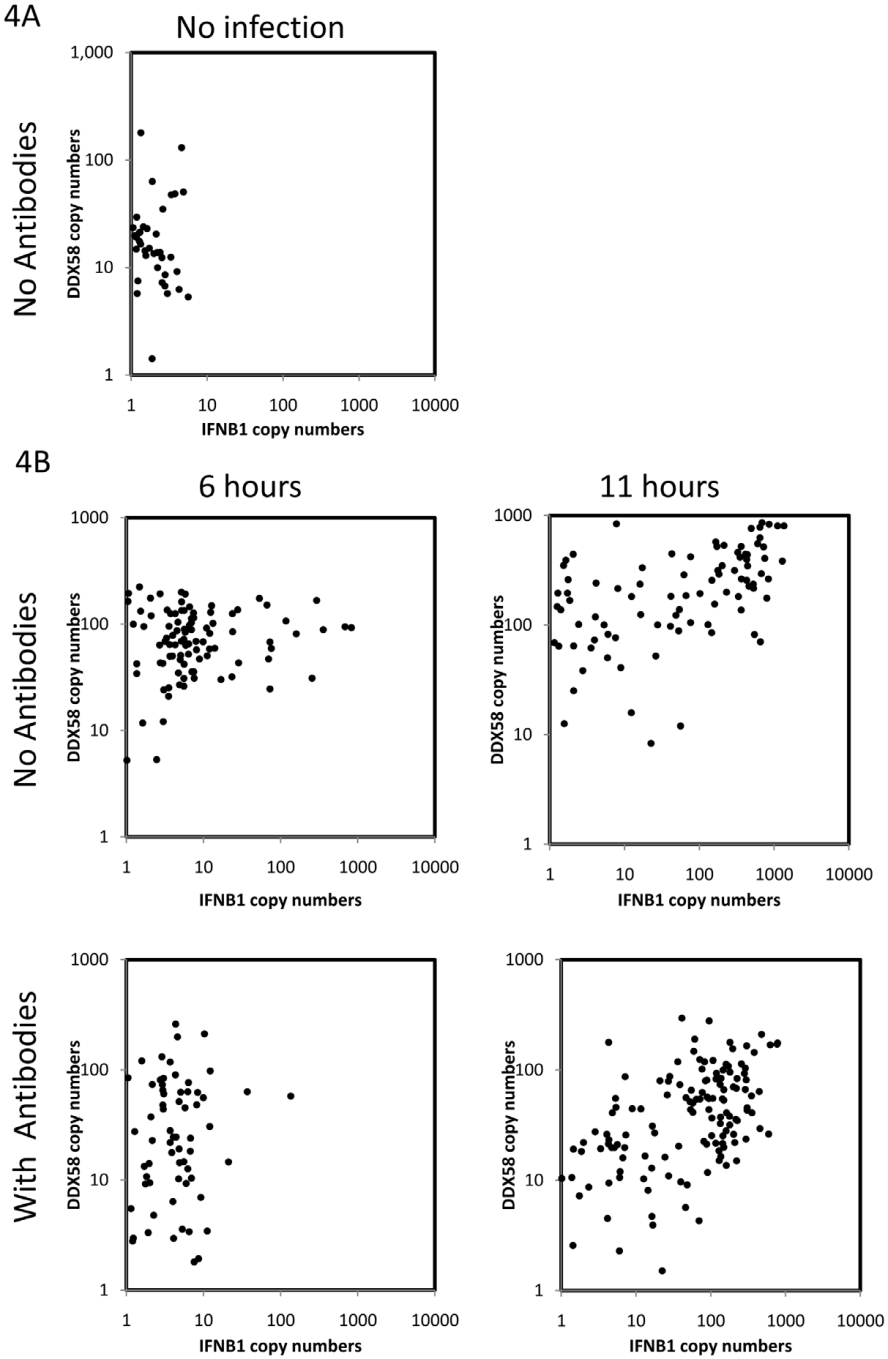
Experimental results in individual DCs with and without blocking antibodies. The scatter plots display *IFNB1* (X axis) and *DDX58* (Y axis) copy numbers in individual cells, as determined by the hemi-nested single cell qRT-PCR, and normalized to *ACTB*. 4A. *IFNB1* and *DDX58* expression in single DCs with no NDV infection and no antibody blockage. 4B. *IFNB1* and *DDX58* expression in single DCs following NDV infection. In the same order as in Figure 3B, experiments without blocking antibodies (upper panels) or with blocking antibodies (lower panels) are shown at 6 hrs (left) and 11 hrs (right) post infection. Note that the dataset for 6 hours was obtained from donor 1, while the datasets for 11 hours and uninfected control were obtained from donor 2.

In order to test the distributions predicted by these simulations, experiments were performed using single cell assays that simultaneously measured both *IFNB1* and *DDX58*. The single cell data presented in **Figure 2B** did not include measurement of both transcripts within each individual cell and could not determine whether the small number of cells showing increased *IFNB1* and *DDX58* levels in the presence of blocking antibodies were the same subset of cells. As had been seen in the simulations, the experimentally obtained co-expression measurements in the presence of blocking antibodies showed a significant correlation between *IFNB1* and *DDX58* (**Figure 4**). Moreover, in the presence of antibodies the level of RIG-I expression in cells drops overall (**Figure 4**), as can also be seen in the simulation results (**Figure 3B**). The interpretation of these experimental results that use antibodies to block interferon signaling is that while the paracrine signaling appears to be efficiently cancelled, the autocrine loop is leaky, leading to the observed correlation and higher copy numbers of *IFNB1* at 11 hours. The persistence of control levels of *DDX58* and of *IFNB1* in some, presumably uninfected, cells at 11 hours when antibodies were present also confirmed the effectiveness of the blockade of paracrine signaling. Overall, the experimental results are in agreement with the salient features about single cell response distribution in the absence and presence of blocking antibodies that were predicted by the model simulations.

**Figure 5:**
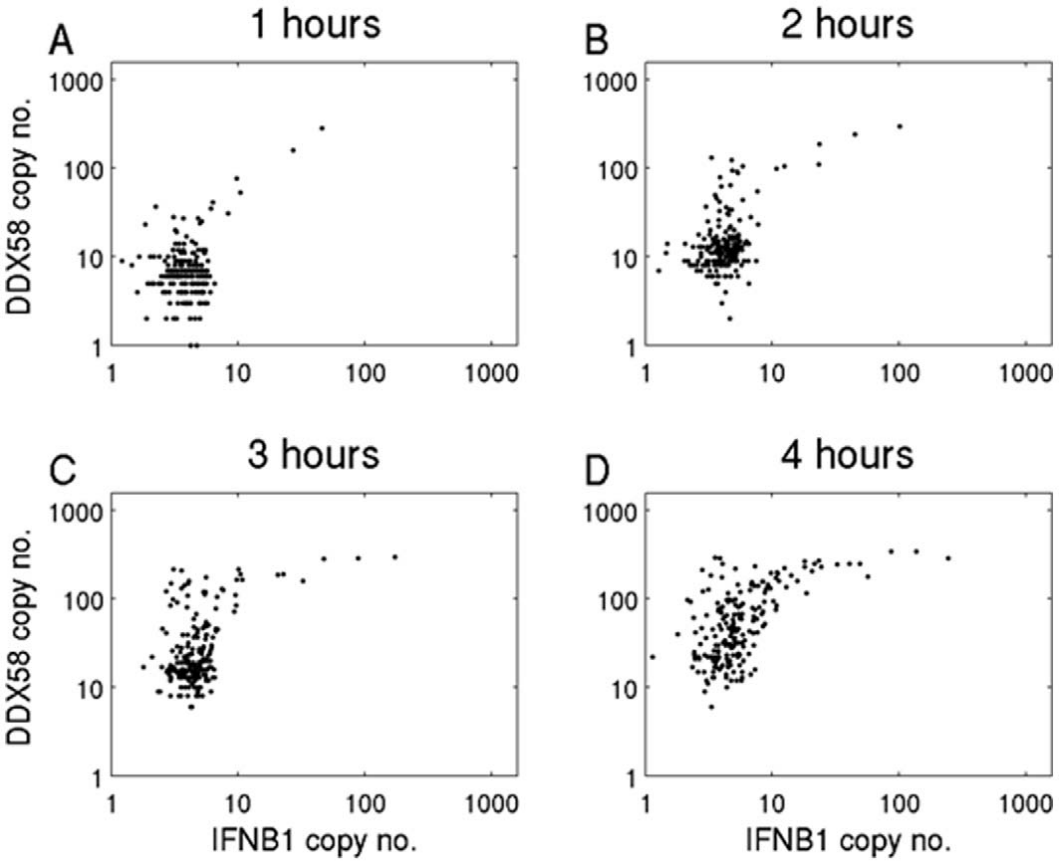
Single cell simulations of early responder DCs. Simulation scatter plots of *IFNB1* (X axis) and of *DDX58* (Y axis) copy numbers in individual DCs at 1, 2, 3 and 4 hours post infection. The simulations were performed with 1000 cells in order to show a sufficient number of cells responding to infection at early time points.

**Figure 6:**
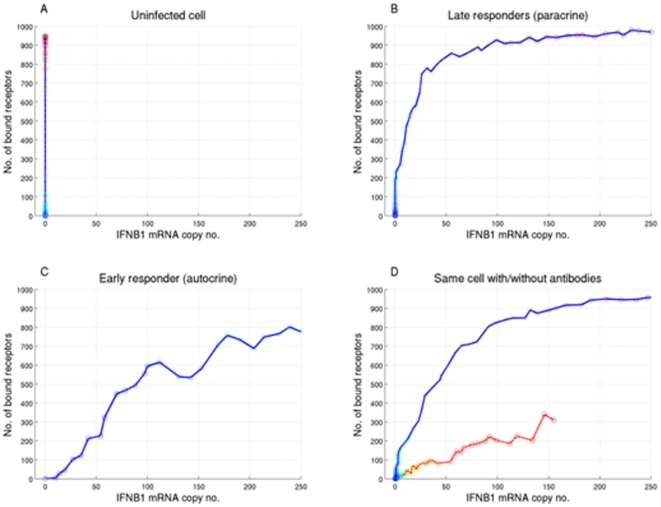
Phase space trajectories of individual cells in simulations. Each plot follows a single cell at 10 minute intervals, and plots the number of bound receptors vs. the number of *IFNB1* messages in that cell at that time point. A. An uninfected cell cannot produce *IFNB1* message, and thus exhibits only an increase in the number of bound receptors. B. A late responder cell is characterized by receptors binding before the cell produces *IFNB1* messages, and is thus activated only through paracrine signaling. C. An early responder cell produces considerable amounts of *IFNB1* message prior to significant receptor binding, suggesting an autocrine activation. The ratio between early and late responders in the simulation is 7∶124. D. A cell that shows a late responder trajectory in the simulation without antibodies (solid line), but changes to a trajectory suggesting autocrine activation when the simulation includes antibodies (dashed line).

This consistency between model and experiment at later times provides some assurance in using simulations to investigate the distribution of responses at early time points. The results of such simulations (**Figure 5**) support the hypothesis that the initiation of the positive feedback loop results from a very small number of cells that are activated and release interferon at early time-points. This is made clear in **Figure 6**, where we plot the number of bound receptors (which is connected to the rate of *DDX58* induction) versus the *IFNB1* copy number for specific cells at different times through the simulation. In the resulting trajectories each point represents the simulation result at time points separated by 10 minutes. Cellular responses fall into typical patterns for uninfected, infected early responder and infected late responder cells. For uninfected cells (**Figure 6A**) there is no *IFNB1* induction despite increasing receptor binding. For early responder cells (**Figure 6C**) there is first *IFNB1* induction followed by increasing numbers of bound receptors and *IFNB1* through early autocrine and later paracrine signaling. For late responder cells (**Figure 6B**) the behavior at early times is opposite to that of early responders with infinite slope for the trajectory instead of zero slope: at first the number of bound receptors increases without any production of *IFNB*1 message, indicating paracrine signaling. Later in the simulation this leads to *IFNB1* production with numbers increasing rapidly through the positive feedback loop. Notably, the ratio between early and late responders is 7∶124 in the simulation, supporting the hypothesis that a small percentage of the population is responsible for activating the whole culture of cells. **Figure 6D** follows the same cell in two simulations, one without antibodies (solid line), and one with antibodies (dashed line), and highlights the effect of suppressing paracrine signaling on late responder cells. In the simulation with no antibodies the cell exhibits a clear late responder trajectory. However, when the paracrine signaling is suppressed the trajectory becomes similar to that of an early responder, where *IFNB1* induction starts before any receptors are bound. This induction occurs late in the simulation and results in lower steady state values, due to partial blocking of the feedback loop.

**Figure 7:**
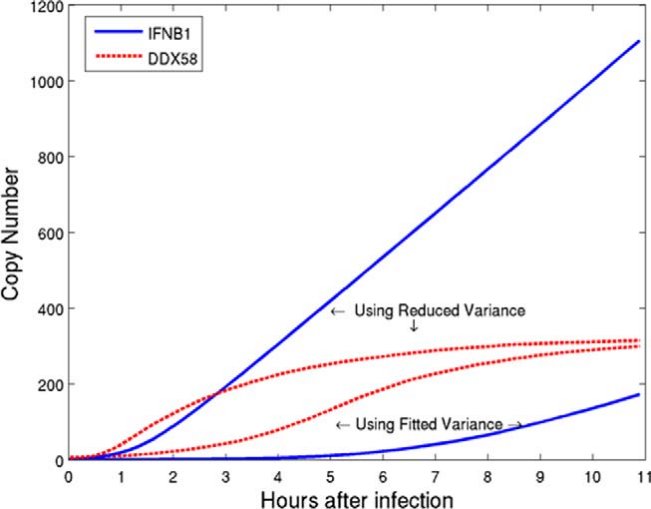
Single DC simulation with high and low cell-to-cell variation. Time course of the average copy number of *IFNB1* per infected cell (solid line) and of *DDX58* (dashed line) obtained from the simulation with the ABM model. The lines marked by “Using Fitted Variance” are drawn from a simulation with parameters fitted to the experimental data, and are identical to the ones shown in Fig. 3A. The lines marked by “Using Reduced Variance” result from a simulation in which the variance of the initial *DDX58* concentration was reduced 10-fold. In order to ensure a similar number of early responder cells, the sensitivity of *IFNB1* to RIG-I concentration was increased by more than 30-fold. The reduction in variability leads to results that do not account for the observed delay in IFN induction relative to DDX58 induction. Furthermore the highly variable system generates low levels of early interferon signaling that can initiate antiviral responses without being prone to later high and potentially toxic levels of interferon secretion.

Thus far we have shown that the proposed mechanism (of a sub population of early responders efficiently activating the rest of the cells) is consistent with the experimental results. Cell-to-cell variations are essential for the process to occur so that interferon-induced genes will seem to be activated prior to significant interferon activation. To test this conjecture, we ran the simulation with decreasing levels of cell-to-cell variability, while maintaining the same average initial RIG-I concentration, and the same percentage of early responding cells To this end, the sensitivity of *IFNB1* induction to the concentration of RIG-I (parameter 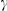) was increased (see Supplementary **Text S2**, Changing the Variance and Maintaining Early Responder Percentage, for details). **Figure 7** shows the original simulation results and the results of a simulation with initial conditions with 10 times less variability in the initial *DDX58* distribution. As the variance is decreased, the sensitivity of *IFNB1* becomes large enough for small fluctuations in the concentration of RIG-I to significantly push a cell closer to activation. As a result, many cells are activated by their internal levels of RIG-I (which is not significantly different from the average) and so the average activation of *IFNB1* occurs earlier. **Figure 7** confirms that decreasing the variability results in similar activation times for *IFNB1* and *DDX58*, supporting our claim that variability is essential in order to induce the dynamics seen in the system. We also note that the simulation with reduced variability results in significantly higher levels of interferon, which can be harmful. As such, the early responder dynamics allows an efficient response to the viral infection while maintaining controlled levels of interferon induction.

## Discussion

The general picture that emerges from our experimental and modeling studies of the early immune response to viral infection provides a fine grain, mechanistic view of how a sub-population of early responder cells can facilitate the activation of a positive feedback loop in the whole population of cells. Two waves of Type I IFN induction by NDV infection of murine fibroblasts were previously observed and attributed to a positive feedback loop of IFNβ and IFNα4 inducing IRF7 [13]. Our single cell level study results are consistent with a positive feedback loop at the population level [13]. At early time points after viral infection, only a few infected cells respond to the infection, while all others remain unaltered. The early responder cells produce and secrete interferon that – after binding to interferon receptors – induces *DDX58* both in themselves and in neighboring cells. If a neighboring cell is already infected, the increased amount of *DDX58* will facilitate viral detection and subsequent *IFNB1* production. This process of *IFNB1* induction is stochastic, depending on many sources of noise, among which are the time to signaling component activation and subsequent time to *IFNB1* enhanceosome formation. Even if a neighboring cell is not infected it will be primed through the induction of *DDX58* to better resist subsequent viral infection. The result is that in all cells *DDX58* is increased beyond the control level (**Figure 2B**) a few hours after infection, while it is only at 6 hours after infection that the full impact of Jak/Stat pathway induction of *DDX58* on *IFNB1* production is seen at the cell population level as the latter rises rapidly on average (**Figure 1A**) with many cells producing a thousand and more copies (**Figures 2** **,** **4**).

The model time behavior allows one to connect the very early cellular responses to experimental measurements at 6 and 10 hours that show a large cell-to-cell variability in interferon induction (**Figures 2** **,** **4**), that is consistent with previous results [14], [15], [16]. The fit to these later time data determines model parameter values, which were then tested by making predictions and comparing them to experiment when interferon diffusion is suppressed. The model shows the right qualitative behavior, which supports the hypothesis that the very early immune response is carried by a small subset of early responder cells. We have thus uncovered in the early immune response another instance where single cell dynamics is different from cell population behavior [17], as has been shown to occur in various systems such as the *Xenopus* oocytes response to progesterone level [18] and PC12 responses to oxidative stress [19]. In view of the experimental difficulty of measuring reliably small numbers of mRNA, our methodology had to rely on a combination of model simulation and experimental validation, with the model providing the bridge between the presumed existence of early responder cells and the later single cell measurements.

One might question some of the simplifying assumptions that went into our model. Firstly, there is the representation in 2 dimensions of 3 dimensional processes. However, since we impose in 2 dimensions average experimental cell distance and diffusion time, we mimic closely in 2 dimensions the temporal 3 dimensional behavior. Thus a crucial aspect of a realistic situation is preserved, despite the fact that in 3 dimensions the number of neighboring cells is larger, which entails that more interferon molecules impinge on a cell's surface receptors. The latter situation can however be compensated for in 2 dimensions by an increase in the receptor binding probability. Secondly, in terms of intracellular processes we limit ourselves to those mRNA that are actually measured, and that compose the *DDX58-IFNB1* feedback loop. We are aware that, although *DDX58* induction plays a role in *IFNB1* induction through its contribution to viral detection, IRF7, which is induced at the same time as RIG-I, is important as well in enhancing interferon production. We concentrated on RIG-I because of its participation in the interferon feedback loop, because *DDX58* induction averaged over the cell population occurs unexpectedly earlier than *IFNB1* induction, and because in the model it appeared reasonable to assume that the distribution of RIG-I across single cells provides the variability that differentiates early from late responders. Eliminating our simplifying assumptions would impose a huge computational burden for the first assumption, and force the introduction of additional reactions and unknown parameters for the second one. We have also performed simulations at lower DC concentration and checked that, although the activation of the late-responding cells was somewhat delayed, the results obtained are qualitatively similar.

In the model, cellular heterogeneity results from a distribution of constitutive RIG-I across cells coupled to a threshold for viral detection. We do not know whether this mechanism is actually the one that distinguishes early responder from late responder cells. We have verified (Supplementary **Figure S3**) that the heterogeneity is not due to the degree of DC differentiation, but other sources of cell-to-cell variability can of course exist.

Our investigation illustrates some of the advantages of integrating experimentation with modeling for immunological studies. Formal modeling sharpens the development of hypotheses and the interpretations of data. It allows rapid computational experiments and leads to predictions to guide subsequent bench experiments. Obtaining representative single cell measurements in individual infected human dendritic cells at early time points is difficult. The experimental intractability of this problem is sharpened by the inability to detect early responder cells even with the extremely sensitive assay that we used. We note that this assay is more accurate than a reporter construct, and is a direct measurement of promoter activity in individual cells. Direct measurement of IFNβ protein production in single cells would be ideal for testing our hypotheses. However, detection of protein level by antibody binding assay would require inhibition of IFNβ secretion, which would alter the DC response to viral infection. The limited detection sensitivity at early time points is also a barrier to precisely measuring the single cell level of IFNβ.

Interestingly, in the key immune response system we have studied, the emergent system-wide behavior results from, and depends on, noise, i.e. the enormous response variability between cells. The noisiness of this system may have physiological importance beyond merely resolving an experimental population level paradox in the *DDX58-IFNB1* loop. The high degree of variation in response and its role in initiating the antiviral feedback loop could play a role in contributing to effective antiviral responses *in vivo* to a wide variety of viral pathogens. The capacity for a few cells to respond early and their capacity to produce an interferon signal that primes other cells could be beneficial in helping mount a response to viruses expressing immune antagonists. Furthermore, the noisiness of the interferon response, in which a few cells are activated but most remain silent, may help in avoiding a cytokine storm while mounting an appropriate response to infection.

## Materials and Methods

### Differentiation of DCs

All human research protocols for this work have been approved by the IRB of the Mount Sinai School of Medicine. The IRB of the Mount Sinai School specifically waived the need for consent due to use of discarded samples not traceable to source. Monocyte-derived conventional DCs were obtained from human blood donors following a standard protocol [20]. Briefly, human monocytes from buffy coats were isolated by Ficoll density gradient centrifugation (Histopaque, Sigma Aldrich) and CD14^+^ monocytes were immunomagnetically purified by using a MACS CD14 isolation kit (Miltenyi Biotech.). CD14^+^ monocytes (0.7×10^6^ cells/ml) were later differentiated into immature MDDCs after 5-6 day incubation in DC growth media [RPMI Medium 1640 (Gibco), 10% fetal calf serum (Hyclone), 2 mM of L-glutamine, 100 units/ml penicillin, 100 µg/ml streptomycin (Pen/Strep) (Invitrogen), 500 U/ml hGM-CSF (Preprotech) and 1000 U/ml hIL-4 (Preprotech)] at 37°C.

### Virus preparation and viral infection

The recombinant Hitchner B1 strain of Newcastle disease virus (rNDV/B1) was prepared as previously described [8], [14]. Using our established protocol [14], the titered NDV stock was diluted 40 times in Dulbecco's Modified Eagle Medium (DMEM) and added directly into pelleted MDDCs at a multiplicity of infection (MOI) of 0.5. MOI was measured as previously described by determining the frequency of cells expressing the NDV L gene mRNA [14]. After incubation for 30 minutes at 37°C, fresh DC growth medium was added back to the infected MDDCs (1×10^6^cells/ml). Virus free allantoic fluid was added to additional tubes of cells to serve as a negative control.

### Antibody blockage

To block IFN signaling, MDDCs (1×10^6^ cells/ml) were pretreated for 30 minutes at 37°C before NDV infection with a cocktail of antibodies including polyclonal sheep anti-human type I IFNα neutralizing antibodies (PBL Biomedical Laboratories) (4000 U/mL), polyclonal sheep anti-human type I IFNβ neutralizing antibodies (PBL Biomedical Laboratories) (4000 U/ml) and monoclonal mouse anti-human type 1 IFN-α/β receptor chain 2 neutralizing antibodies (Antigenix) (10 µg/ml).

### Sorting of single MDDCs

Single MDDCs were directly sorted into 384-well PCR plates as previously described [14]. Briefly, MDDCs were screened and sorted by visual light scatter or labeled fluorescence (MoFlo high speed cell sorter) directly into 384-well bar-coded PCR plates (Roche LC480), which contained 5 µL cell lysis buffer [4 mM magnesium acetate (Sigma), 0.05% NP40 (Sigma), 0.8 U/µL Protector RNAse Inhibitor (Roche Applied Sciences)] in each well. Sorted MDDCs were immediately placed on dry ice and stored at –70°C to prevent RNA degradation.

### Real time RT-PCR of total RNAs

Cultured human MDDCs (1×10^6^cells) were divided into two samples (5×10^5^cells each). Cells in one sample were infected by NDV at an MOI of 0.5 and cells in another sample were uninfected and used as an experimental control. Total RNAs were isolated from both samples using an RNeasy mini kit (Qiagen) after 10-hour infection. 100 ng of RNA sample was used as template for real time RT-PCR to determine the expression levels of *IFNB1* and *DDX58*. The details of the PCR reactions are presented in Supplementary **Text S1** (Detailed PCR Protocols). The data were normalized to the housekeeping gene *ACTB*.

### Single cell hemi-nested PCR

Single MDDCs were sorted directly into 384-well bar-coded PCR plates as previous described [14]. Specifically, a 5 µL aliquot of a 2x AccuRT master mix solution prepared as described above was added to each well. In control wells with no cells, 1 µL of genomic DNA of varying dilutions (10^3^–40 copies/µL) was added along with the 5 µL aliquot of 2x master mix. Hemi-nested PCR was performed as described in Supplementary **Text S1** (Detailed PCR Protocols) for the multiplex hemi-nested PCR of *IFNB1* and *DDX58*. PCR results were analyzed with the Roche Lightcycler 480 where the PCR cross point (Cp) value for each amplification curve was determined by a secondary derivative calculation. Cp values were converted to copy numbers using the absolute quantification method based on Cp values for the genomic DNA standards. An illustration of the experimental procedure is shown in Supplementary **Figure S4** with validation data in Supplementary **Figures S5,S6**. A few PCR dropouts (for both *IFNB1* and *DDX58*) were observed and excluded from analysis.

### Agent based model of RIG-I mediated DC response to viral infection

Single-cell stochastic behavior is important in many biological processes, leading to phenotypic variability among genetically identical organisms and determining cellular fate following viral infection in bacteria and eukaryotic cells [21], [22], [23], [24], [25], [26], [27], [28], [29]. In order to test the hypothesis of early responder cells we developed an agent based model (ABM) that simulates the *IFNB1*-*DDX58* positive feedback loop. The simulation emulates a portion of a medium containing both infected and uninfected MDDCs. The inter-cellular model we use is two dimensional (2d), and the medium is represented by a square lattice, where each lattice square has the size of a single cell (Supplementary **Figure S7**). Each cell is simulated as an independent agent, where the agents interface through the extracellular medium. The graphical display engine generates an animation by depicting the state of the simulated area at each simulation step.

Where possible, parameters used in the simulation were based on experimental results. The experimental basis for parameter values related to extracellular signaling was summarized previously [30]. We assume that these same values are applicable to our 2d lattice model. The measured diffusion rate is of the order of 
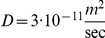
, and the diameter of a cell is 30 µm. The diffusion coefficient and the lattice unit size determine the time step in the simulation, leading to a time step of 9 seconds (as explained below). We chose the total lattice size according to the diffusion distance after 11 hours, which is the latest measurement time. Since the average displacement of a molecule performing a random walk or diffusing increases like the square root of time this leads to a lattice size of 40 by 40 units. According to [30], 6.5% of the medium's volume is occupied by the cells, which translates to an average distance between neighboring cells of about 2.5 times their diameter. We used this average distance between nearest neighbors in the 2d simulation, which leads to a population of a little over 200 cells in the whole lattice, distributed randomly.

### Intra-cellular simulation

In order to reflect the variations in response that result from a small number of reactants within individual cells, the dynamics of each cell was simulated using a stochastic simulation based on Gillespie's algorithm. The simulation follows the number of transcripts of *IFNB1* and of *DDX58*, the number of RIG-I proteins, and the number of bound receptors on every cell. The initial RIG-I level in each cell was chosen from a log-normal distribution so that a small number of cells had a large amount of RIG-I, while the majority had a small amount [31]. The parameters for *IFNB1* induction were chosen so that only cells with sufficient RIG-I could be induced. RIG-I concentration in infected cells determines *IFNB1* induction in a Michaelis-Menten like form. Bound cell surface receptors activate *DDX58* transcription according to a Michaelis-Menten like function.

### Stochastic Processes, Rates, and Rate Constants

**Figure 8:**
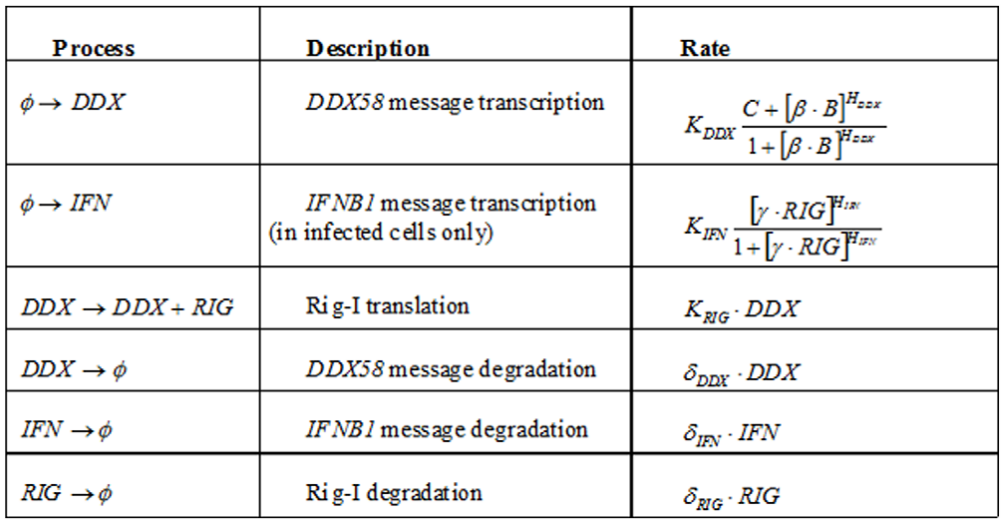
Processes, Descriptions and Rates. The processes, descriptions and rates for each of the six reactions in the stochastic simulations.

The modified Gillespie algorithm to stochastically simulate each cell follows the time dependence of *IFNB1* transcripts, *DDX58* transcripts, and RIG-I proteins. These molecular species are denoted as IFN, DDX, and RIG, respectively. The simulation follows six reactions, as summarized in Figure 8: *DDX58* message transcription, *IFNB1* message transcription, RIG-I translation, *DDX58* message degradation, *IFNB1* message degradation, and RIG-I degradation.

The rate constants for IFN and DDX transcription are denoted by 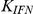 and 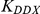, respectively. The translation rate constant for RIG is given by 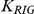. The degradation rate constants for IFN, DDX, and RIG are given by 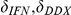, and 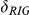, respectively. The transcription rate *DDX58* depends on the number of bound interferon receptors, denoted by B, in a Michaelis-Menten form using a Hill coefficient of 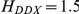 and a half-induction level given by 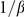 (meaning that when 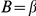, *DDX58* reaches half it maximal induction). According to experiments, *DDX58* is constitutively expressed in the cells. The ratio between the maximal induction of *DDX58* and the constitutive induction is given by 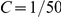. *IFNB1* induction depends on Rig-I concentration in a Michaelis-Menten form with a Hill coefficient 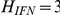, and a half-induction concentration give by 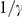. All the degradation processes follow an exponential decay. The processes and the rates at which they occur are summarized Figure 8 (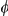 indicates a source or a sink, used to denote creation or degradation of molecules, respectively):

The values used for the rate constants are based on experimental results, and were changed to fit the experimental data. Specifically, in the simulation results shown in the paper the values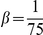, 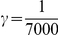, 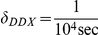, 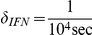, and 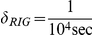 were used. In order to account for some of the cell-to-cell variation, the transcription rate constants for each cell in the simulation were chosen from a Gaussian distribution around the values 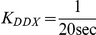, 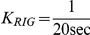, with standard deviations of 0.01, and values below 0.01 and above 0.05 were ignored and re-sampled.

### Inter-cellular Diffusion Simulation

The inter-cellular medium is simulated by a Monte-Carlo simulation on a 2d square lattice. The simulations were performed in 2D to reduce computational overhead and because, as discussed below, the conclusions are applicable to the 3D case. The inter-cellular simulation follows the diffusion of interferon molecules in the medium, which is described by the diffusion equation. Thus the average displacement of each molecule from its origin increases as the square root of time. This result holds for 3d systems, as well as for 2d ones. The difference between 2D and 3D can be accounted for by a change in the value of the diffusion coefficient. In order to preserve the average time for the diffusion to a neighboring cell, we keep the average distance between neighboring cells equal to the one found in 3d. Besides the distance between nearest neighbors, the interaction between secreted interferon molecules and a neighboring cell also depends on the probability of binding to cell-surface receptors, and the dimensionality of the system. By changing the cell surface binding probability, we can replace the 3d system by an equivalent 2d system, in which the interaction of neighboring cells is unchanged. We recognize that there are differences between the 2D and 3D systems. For example, in random walks in 2d as compared to 3d, the probability of a molecule to return to its origin is higher. Therefore, in our simulations, the amount of paracrine signaling is underestimated compared to autocrine signaling. However, this difference is immaterial for the questions addressed and for the conclusions of the present study.

The 2d lattice is simulated using periodic boundary conditions, and its grid size is set to be the diameter of a single cell, denoted L, which is approximately 30 µm. Cells are distributed inside the matrix randomly with a density that ensures the correct average distance to nearest neighbors (as explained above). Each cell has a probability of 50% of being infected or uninfected, in accordance with the experimental MOI of 0.5. Uninfected cells do not transcribe interferon. At each grid point the simulation follows the amount of interferon molecules, denoted *I*, and uses the number of free and bound interferon receptors obtained from each cell, denoted *F*, and *B*, respectively. Grid squares that do not contain a cell have zero *F* and *B* at all times. Receptor binding and unbinding by IFNβ are adapted from work described in reference [30].

At each time step interferon molecules in lattice squares containing cells may bind to free surface receptors, while bound receptors may unbind. The binding rate is given by 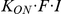, where 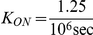 is the binding rate constant. The unbinding rate constant was found to be 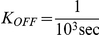. The rates per receptor are slow enough so that for the chosen time-step we have 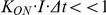 and 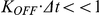. Therefore we approximate the binding probability of each free receptor in a time step by 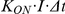, and the unbinding probability for every bound receptor by 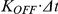.

The distribution of molecules in a diffusion-governed system with initial condition of all the molecules concentrated at the origin is expressed by a Gaussian function 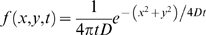, allowing calculation of the time by which half the molecules pass a distance of a cell radius, giving approximately 9 seconds (solving the equation 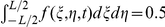, with *L* = 30 µm). We use this time interval as the basic time-step in the simulation, during which each interferon molecule has a probability of 50% to pass to a neighboring lattice square. As a result, in each simulation step each interferon molecule in each cell has a probability of 50% of moving to one of the four neighboring cells. This ensures a random walk behavior for a small number of molecules, and a diffusion behavior for large numbers of molecules.

### Synchronization Between Inter and Intra-Cellular Simulation

The Monte-Carlo simulation and the Gillespie simulation run at different time-scales and different length scales. The spatial interaction is explicitly manifested by assigning each cell to a unique lattice square. In order to account for the different time scales we must synchronize the constant time step of the Monte-Carlo with the variable (and often much faster) time step of the Gillespie simulations. To do this, we allow the Gillespie algorithm of each cell to run for a number of steps, until the total amount of time of these steps exceeds the Monte-Carlo time step. The last step is not performed, and the time is advanced to match the next Monte-Carlo time step. This procedure ensures the simulation of a real Markovian process when the external conditions change at pre-determined times.

Another aspect of synchronizing the inter-cellular Monte-Carlo simulation with the intra-cellular Gillespie simulations involves the secretion of interferon molecules from each cell. At each Monte-Carlo step, each cell is queried for the number of *IFNB1* messages in that cell. We assume that the number of new interferon proteins at each simulation time step is the number of *IFNB1* transcripts multiplied by a factor of M = 0.1 (which corresponds to a translation rate of 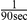). We further assume that interferon secretion is a rapid process and that all the newly synthesized interferon is secreted at each time step. The number of secreted interferon molecules is added to the number of interferon molecules in the lattice square occupied by the cell.

### Simulations with Interferon-Blocking Antibodies

Two kinds of antibodies were introduced in the experiments. One targets the interferon molecules, and the other targets the interferon cell surface receptors. To include this in the simulation we added two parameters to the Monte-Carlo simulation of the inter-cellular medium. First we allowed each interferon molecule to become inactive by binding to an antibody with a probability of 0.4 at each Monte-Carlo time step. This corresponds to an effective degradation rate of approximately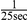, and allows the interferon molecules to form a cloud around the emitting cells, but hardly any can reach a neighboring cell. The binding rate was reduced by multiplication of 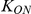
by a factor of 0.9. This means that even the few molecules that are able to reach neighboring cells have a harder time activating it, and is equivalent to reducing the number of active receptors by the same factor of 0.9.

## Supporting Information

Figure S1 Measurement of NDV L gene, *IFNB1*, *IFNA1*, *MXA*, and *DDX58* transcripts in human MDDCs at 0, 3, 6 and 10 hours following NDV infection. Total mRNA expression of these genes was quantified by real-time RT-PCR and induction fold increases were normalized to the initial value at 0 hrs post infection. A. *IFNB1*; B. *DDX58*; C. NDV L gene; D. *MXA*; E. *IFNA1.*
(TIF)

Figure S2 Time course for *IFNB1* and *DDX58* induction in MDDCs infected by NDV at different MOI. MDDCs were infected by NDV at MOI = 0.1, 0.5, 1.0, 2.0 and 5.0. Expression levels were measured at 1, 2, 3, 4, 5, 6 and 10 hours after infection. The relative induction level was normalized to *ACTB* and to an uninfected control. A. *IFNB1* (upper panel early times, lower panel later times) B. *DDX58*.
(TIF)

Figure S3 Effect of DC heterogeneity in the DC response to NDV infection. The real-time RT-PCR cross point (Cp) value indicates the level of *IFNB1* in each individual DC (high Cp value means low expression). 6 hour after NDV infection, single MDDCs sorted by DC differentiation marker CD14 (0 =  CD14+ high, 1 = CD14+ low) showed high heterogeneity in both groups but no significant difference in *IFNB1* production between the two groups (p = 0.17).
(TIF)

Figure S4 Schematic illustrating the hemi-nested PCR protocol used to measure *IFNB1* mRNA levels in single DCs. DCs were directly sorted into 384-well PCR plates prepared with lysis buffer. Amplification of mRNAs was performed with the two-step hemi-nested real time RT-PCR. The first step was reverse transcription reaction followed by 8-cycle amplification. Internal control oligonucleotides containing mutations (AA/TT substitution and deletion of the Roche LNA probe binding site) and *IFNB1* mRNA transcripts were both amplified by the primers in this first step reaction. The PCR products were split into two PCR wells and further amplified and detected by second real time PCR reactions. An *IFNB1* cDNA-specific forward primer with AA at the 3′end and the *IFNB1* cDNA-specific Roche LNA Taqman probe were added into the second real time PCR reaction to discriminate the PCR products from internal control oligonucleotides. The final amplification signal of the second PCR originated only from the *IFNB1* mRNAs.
(TIF)

Figure S5 Validation of the hemi-nested real time PCR method with serial dilutions of genomic DNA. 5A. Human genomic DNA was used as the PCR template and serial diluted to 4000 copies, 400copies, 40 copies, 4 copies in each well. The PCR amplification curves of 4 repeats of 4000 copies and 400 copies, 8 repeats of 40 copies, 10 repeats of 4 copies and negative controls are shown. A standard curve of qcPCR with diluted genomic DNA is presented as an inset. The linear fitting equation and r^2^ shown in the inset were given by KaleidaGraph. 5B. Real-time PCR amplification curve of human genomic DNA standard using *IFNB1* control oligonucleotide specific primer. This oligonucleotide specific primer has been designed to anneal only with PCR amplicons originated from the *IFNB1* control oligonucleotide (details see **Materials and Methods** section). Our data showed a very tight distribution at the different concentrations of the genomic standard (10^3^–fold range).
(TIF)

Figure S6 Validation of multiplexed hemi-nested real time PCR detection with total RNA dilutions. Total RNAs were extracted from MDDCs and 10-fold serially diluted until the final concentration reached copy numbers similar to the low copy genomic DNA standards depicted in Supplementary **Figure S5A**. In order of RNA copy number from high to low, the results are for results from 6 repeats, 6 repeats, 12 repeats and 18 repeats of PCR reactions. 6A. PCR amplification curve of *IFNB1* for total RNA dilutions. 6B. PCR amplification curve of *DDX58* for total RNA dilutions. The final dilution did not show *DDX58* amplification due to lower expression of *DDX58* than *IFNB1* (less than 1 copy/cell).
(TIF)

Figure S7 Two dimensional agent-based model (ABM). The extracellular model is two dimensional, and the medium is represented by a square lattice, where each lattice square has the size of a single cell. Each cell is simulated as an independent agent, where the agents interface through the extracellular medium.
(TIF)

Text S1 Detailed PCR Protocols.
(DOCX)

Text S2 Changing the Variance and Maintaining Early Responder Percentage.
(DOCX)
