# Supplementary figures and images for: Role of Cell-to-Cell Variability in Activating a Positive Feedback Antiviral Response in Human Dendritic Cells
Source: PLoS One. 2011 Feb 8;6(2):e16614. doi: 10.1371/journal.pone.0016614 (PMC3035661; doi:10.1371/journal.pone.0016614)

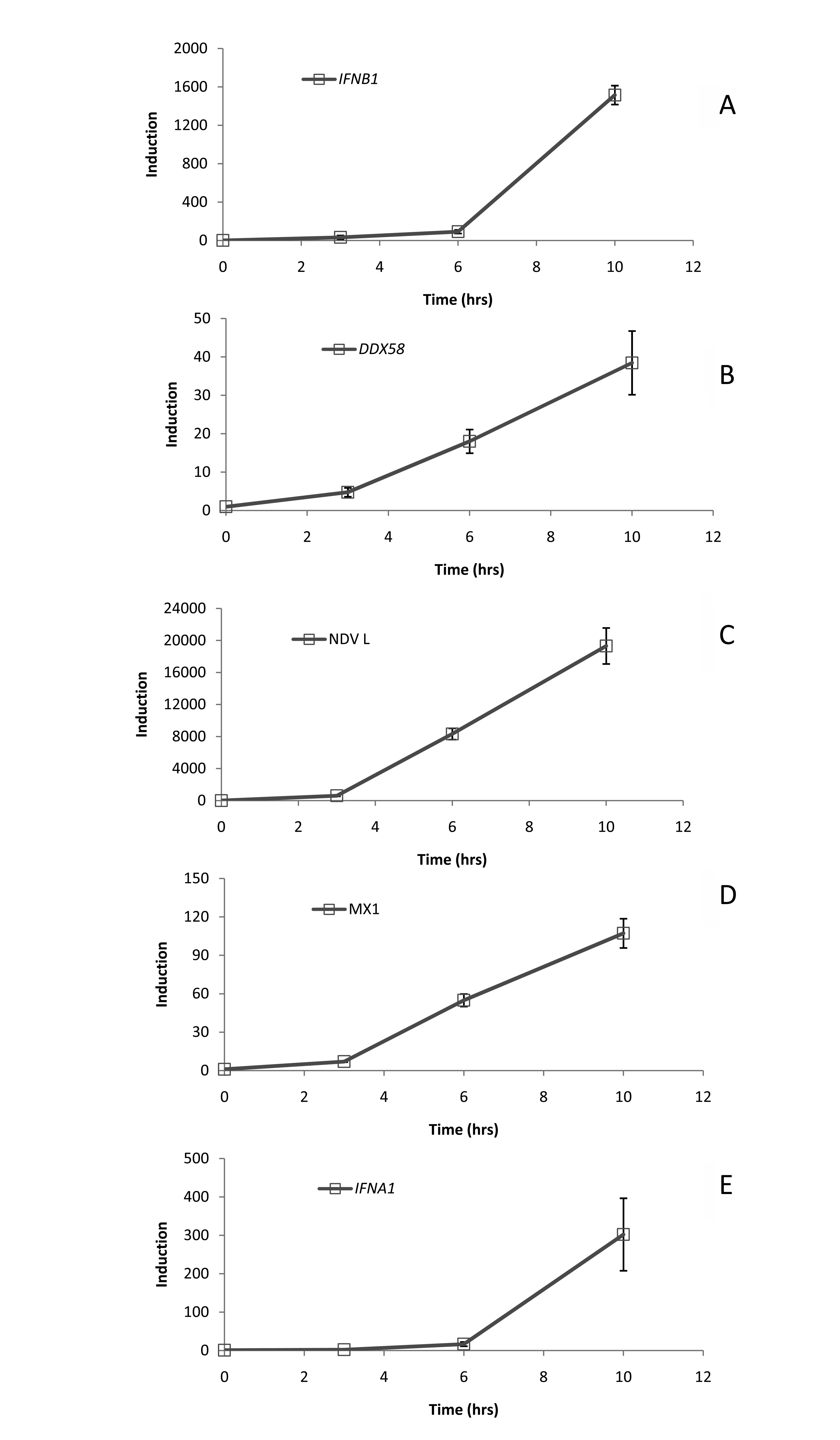

Supplement: Figure S1 — Measurement of NDV L gene, IFNB1, IFNA1, MXA, and DDX58 transcripts in human MDDCs at 0, 3, 6 and 10 hours following NDV infection. Total mRNA expression of these genes was quantified by real-time RT-PCR and induction fold increases were normalized to the initial value at 0 hrs post infection. A. IFNB1; B. DDX58; C. NDV L gene; D. MXA; E. IFNA1. (TIF) [file pone.0016614.s001.tif]

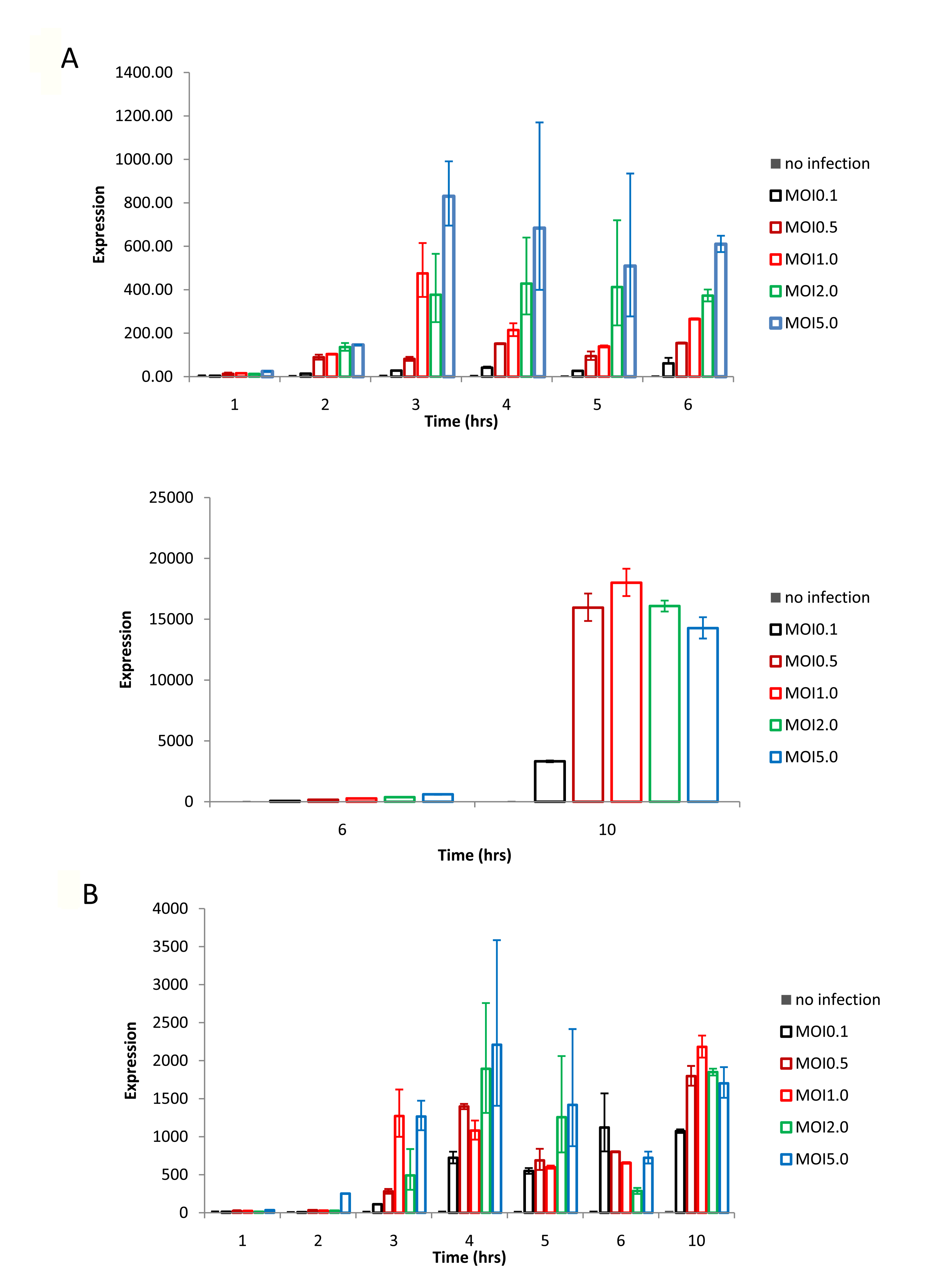

Supplement: Figure S2 — Time course for IFNB1 and DDX58 induction in MDDCs infected by NDV at different MOI. MDDCs were infected by NDV at MOI = 0.1, 0.5, 1.0, 2.0 and 5.0. Expression levels were measured at 1, 2, 3, 4, 5, 6 and 10 hours after infection. The relative induction level was normalized to ACTB and to an uninfected control. A. IFNB1 (upper panel early times, lower panel later times) B. DDX58. (TIF) [file pone.0016614.s002.tif]

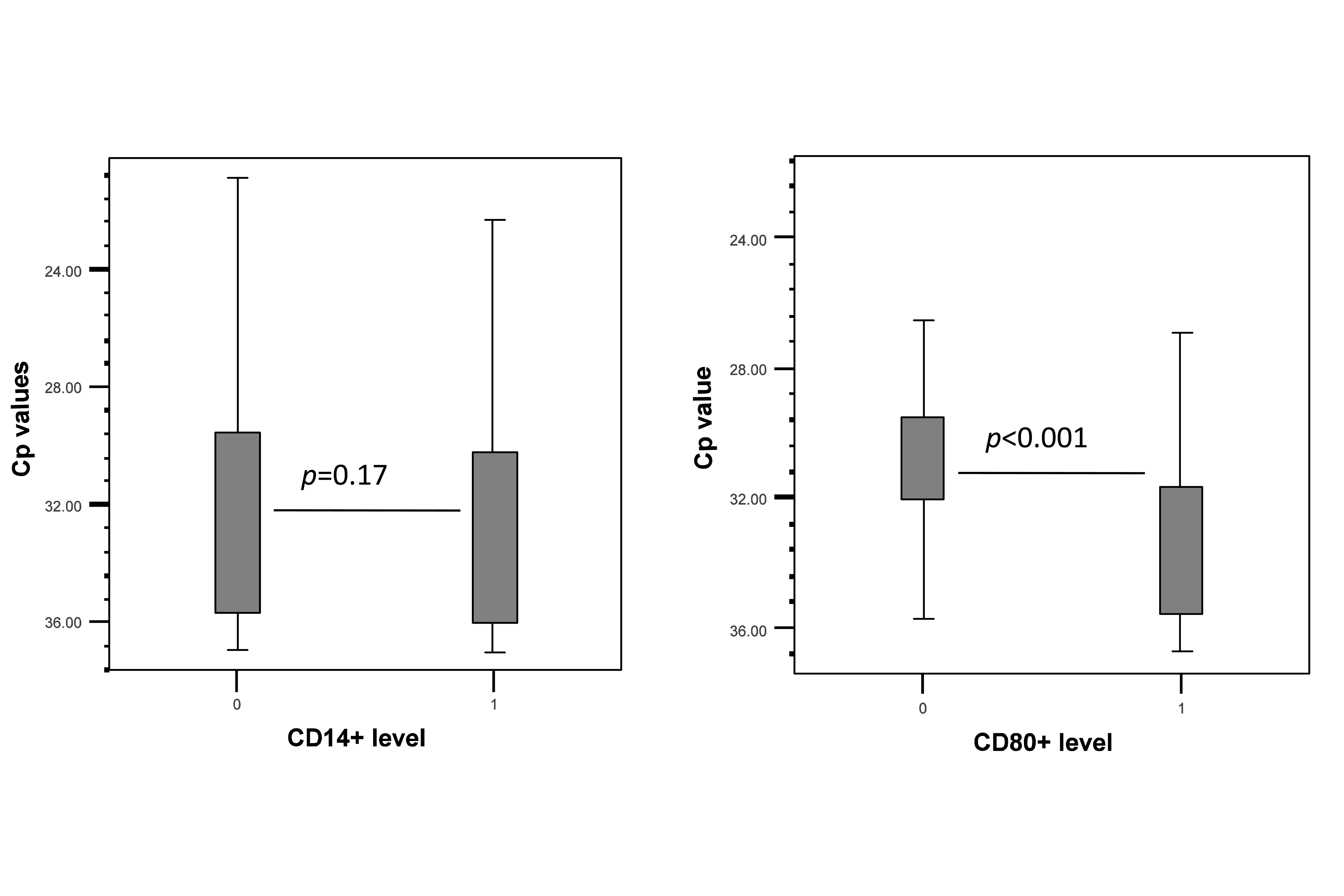

Supplement: Figure S3 — Effect of DC heterogeneity in the DC response to NDV infection. The real-time RT-PCR cross point (Cp) value indicates the level of IFNB1 in each individual DC (high Cp value means low expression). 6 hour after NDV infection, single MDDCs sorted by DC differentiation marker CD14 (0 = CD14+ high, 1 = CD14+ low) showed high heterogeneity in both groups but no significant difference in IFNB1 production between the two groups (p = 0.17). (TIF) [file pone.0016614.s003.tif]

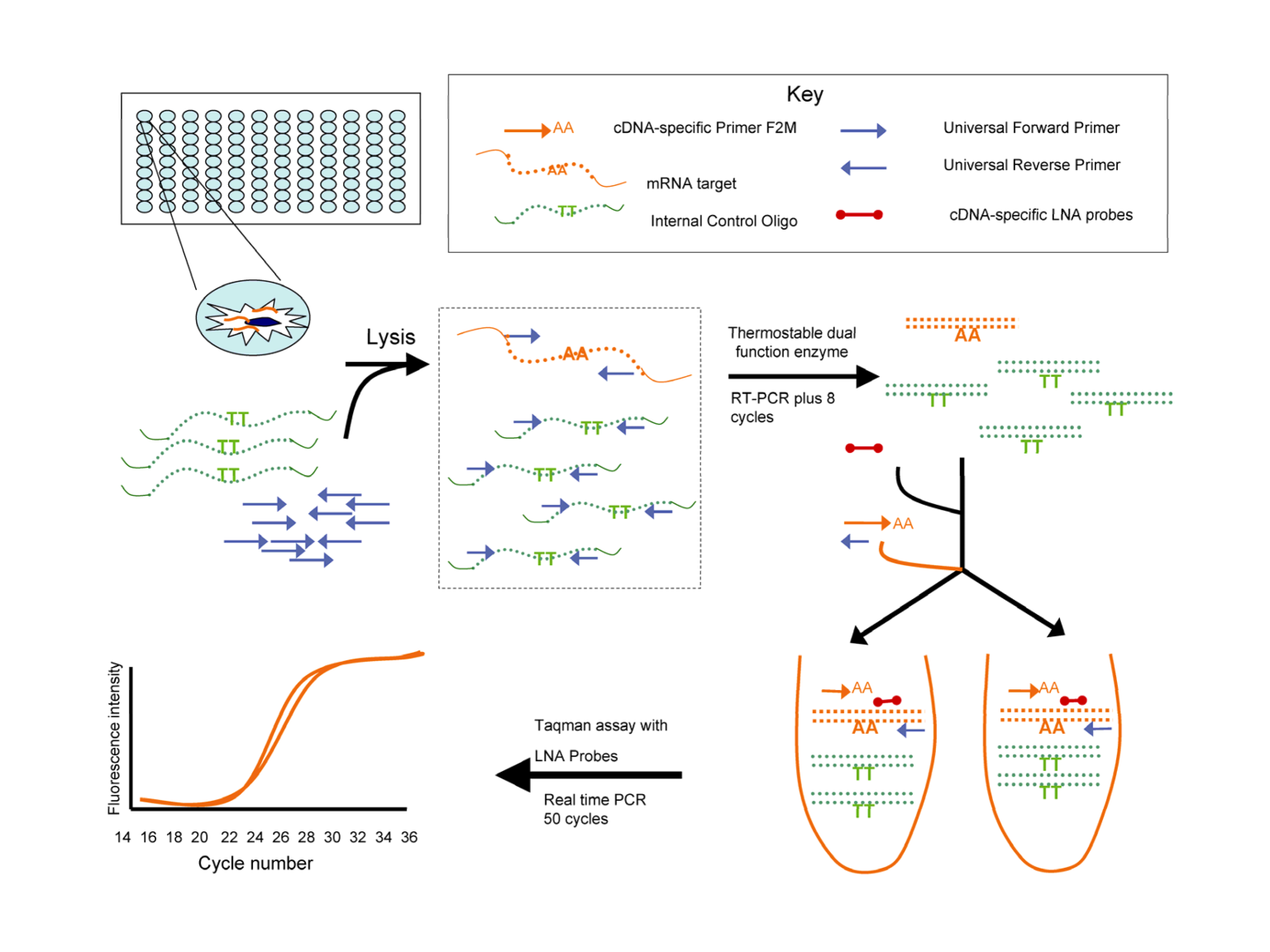

Supplement: Figure S4 — Schematic illustrating the hemi-nested PCR protocol used to measure IFNB1 mRNA levels in single DCs. DCs were directly sorted into 384-well PCR plates prepared with lysis buffer. Amplification of mRNAs was performed with the two-step hemi-nested real time RT-PCR. The first step was reverse transcription reaction followed by 8-cycle amplification. Internal control oligonucleotides containing mutations (AA/TT substitution and deletion of the Roche LNA probe binding site) and IFNB1 mRNA transcripts were both amplified by the primers in this first step reaction. The PCR products were split into two PCR wells and further amplified and detected by second real time PCR reactions. An IFNB1 cDNA-specific forward primer with AA at the 3′end and the IFNB1 cDNA-specific Roche LNA Taqman probe were added into the second real time PCR reaction to discriminate the PCR products from internal control oligonucleotides. The final amplification signal of the second PCR originated only from the IFNB1 mRNAs. (TIF) [file pone.0016614.s004.tif]

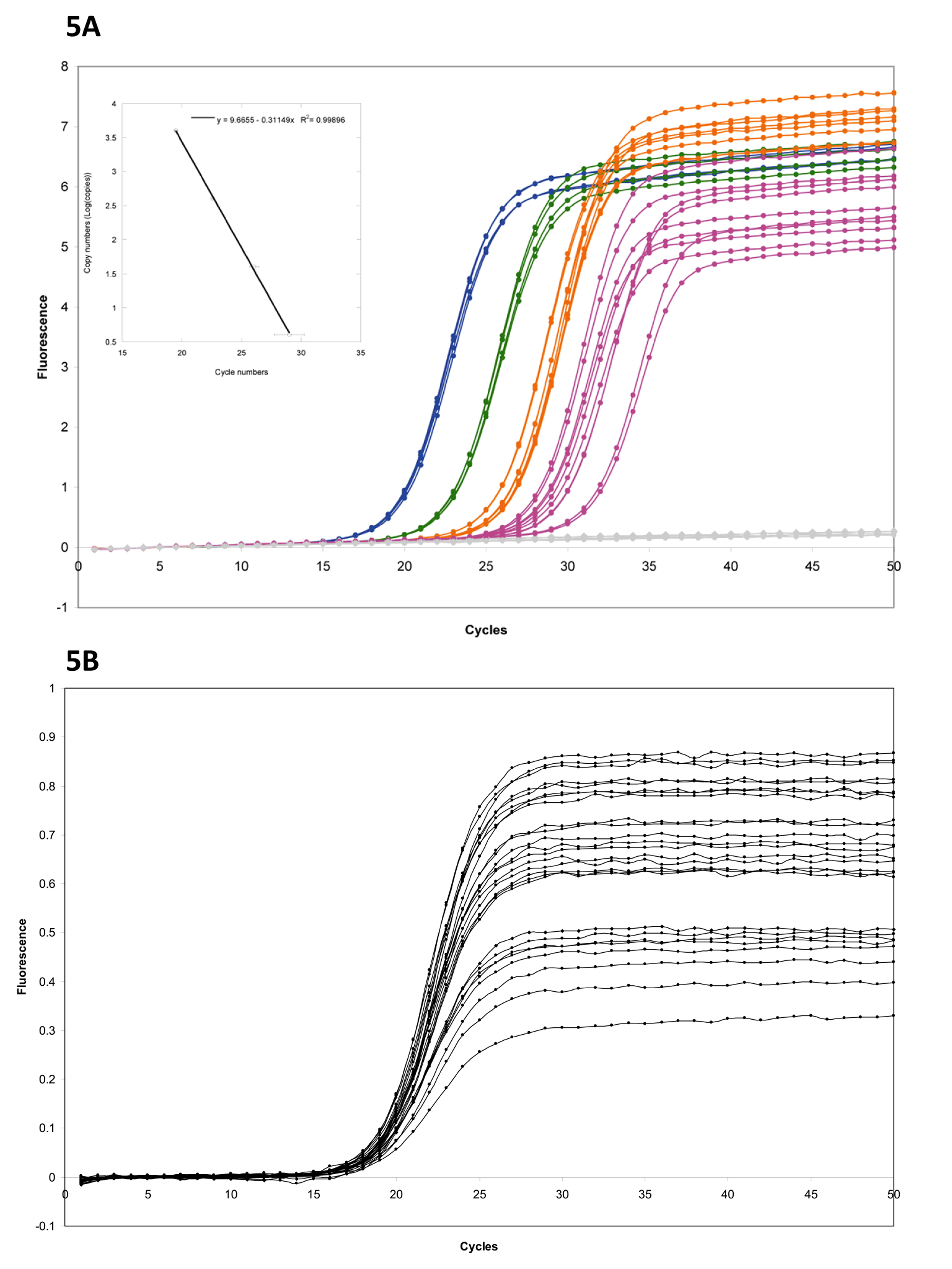

Supplement: Figure S5 — Validation of the hemi-nested real time PCR method with serial dilutions of genomic DNA. 5A. Human genomic DNA was used as the PCR template and serial diluted to 4000 copies, 400copies, 40 copies, 4 copies in each well. The PCR amplification curves of 4 repeats of 4000 copies and 400 copies, 8 repeats of 40 copies, 10 repeats of 4 copies and negative controls are shown. A standard curve of qcPCR with diluted genomic DNA is presented as an inset. The linear fitting equation and r2 shown in the inset were given by KaleidaGraph. 5B. Real-time PCR amplification curve of human genomic DNA standard using IFNB1 control oligonucleotide specific primer. This oligonucleotide specific primer has been designed to anneal only with PCR amplicons originated from the IFNB1 control oligonucleotide (details see Materials and Methods section). Our data showed a very tight distribution at the different concentrations of the genomic standard (103–fold range). (TIF) [file pone.0016614.s005.tif]

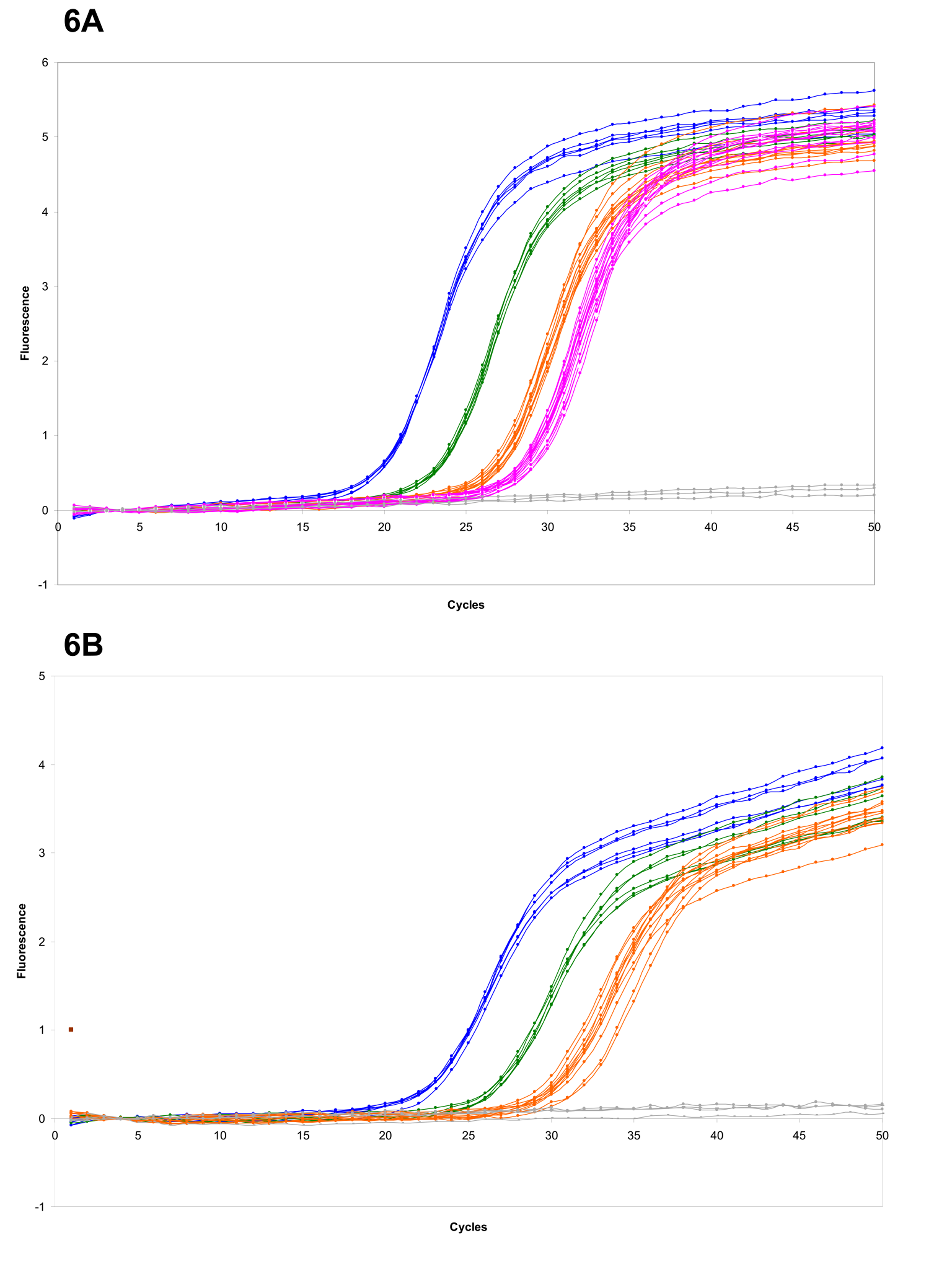

Supplement: Figure S6 — Validation of multiplexed hemi-nested real time PCR detection with total RNA dilutions. Total RNAs were extracted from MDDCs and 10-fold serially diluted until the final concentration reached copy numbers similar to the low copy genomic DNA standards depicted in Supplementary Figure S5A. In order of RNA copy number from high to low, the results are for results from 6 repeats, 6 repeats, 12 repeats and 18 repeats of PCR reactions. 6A. PCR amplification curve of IFNB1 for total RNA dilutions. 6B. PCR amplification curve of DDX58 for total RNA dilutions. The final dilution did not show DDX58 amplification due to lower expression of DDX58 than IFNB1 (less than 1 copy/cell). (TIF) [file pone.0016614.s006.tif]

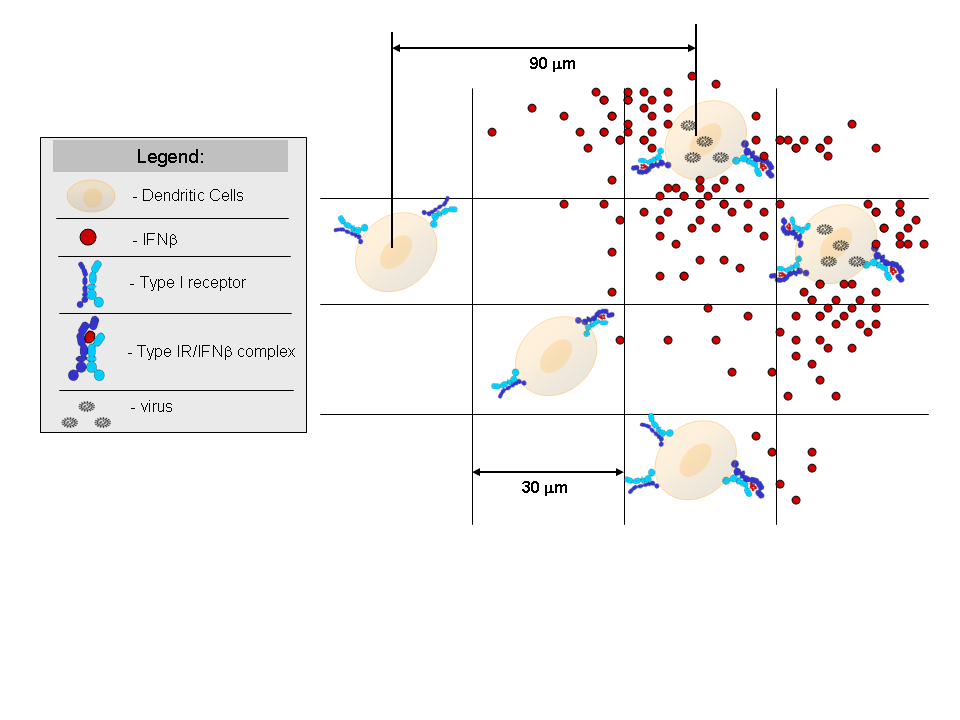

Supplement: Figure S7 — Two dimensional agent-based model (ABM). The extracellular model is two dimensional, and the medium is represented by a square lattice, where each lattice square has the size of a single cell. Each cell is simulated as an independent agent, where the agents interface through the extracellular medium. (TIF) [file pone.0016614.s007.tif]
