# Supplementary material for: Role of Cell-to-Cell Variability in Activating a Positive Feedback Antiviral Response in Human Dendritic Cells
Source: PLoS One. 2011 Feb 8;6(2):e16614. doi: 10.1371/journal.pone.0016614 (PMC3035661; doi:10.1371/journal.pone.0016614)
Supplement: Text S1 — Detailed PCR Protocols. (DOCX) [file pone.0016614.s008.docx]

## Supplementary Text S1: Detailed PCR Protocols

### Oligonucleotides and primers design

Regular RT-PCR primers used for quantification of *ACTB*, *DDX58* and *IFNB1* in total RNA samples were described previously [13]. For hemi-nested real time RT-PCR, the primers were redesigned to be compatible with the Roche Universal Probe Library (UPL). Sequences of the *IFNB1* and *DDX58* primers and control oligonucleotides (IDT Technologies) and LNA probes (Roche Applied Science) for human single cell assays are given in the table below. The primer sequences for the intron-containing genes *DDX58* and *ACTB* were derived from adjacent exons. Our multiplex transcription measurement uses sequential PCR reactions. Forward and reverse primers for the first PCR were designed to anneal to and amplify both the cDNA template and spiked control oligonucleotides. Internal control oligonucleotides for *IFNB1* and *DDX58* were designed to be identical to the cDNA sequence but with a 2nt substitution replacing 8nt including the 6nt Roche LNA probe binding site to prevent extension by either allele-specific primer and to prevent probe binding and detection. PCR Primers were dissolved in DEPC-treated H_2_O at 20 µM. Control oligonucleotides were stocked at 20 µM in DEPC-treated water with 20 ng/µL Polyinosinic Acid (Sigma-Aldrich). In the second PCR, allele specific forward primers were designed to anneal with only the cDNA sequence.

### Hemi-nested PCR

Measuring gene expression in single cells required a quantitative multiplex method for quantifying transcripts at low copy numbers. Nested PCR has been found to improve PCR sensitivity and specificity by selecting against non-specific priming. We have extended the technology of our previous single cell studies [13] by using hemi-nested single cell PCR with locked nuclear acid (LNA) Taqman detection to quantify low copy numbers of *IFNB1* and *DDX58* transcripts in the same single human DCs. An illustration of the protocol is shown in Supplementary **Figure S4**.

### Validation of hemi-nested PCR method

To evaluate the sensitivity of the hemi-nested PCR, human genomic DNA (Promega) was serially diluted in DEPC treated water with 20ng/μl Poly I, containing 2000, 200, 20, 2 and 0 copies of DNA in 1μl. The first 10μl PCR reaction contained 5μl of 2x Roche Lightcycler Probe Master Mix (with polymerase), 0.1µM final concentration of universal primer sets for *IFNB1*, control *IFNB1* oligonucleotides at 200 copies, and 2μl of template genomic DNA standard. Real time PCR was performed in a Roche Lightcycler 480. The samples were amplified according to the following procedure: polymerase activation at 95°C for 10 minutes, followed by 8 cycles at 94°C for 10s, 60°C for 15s, and 72°C for 15s. PCR without control oligonucleotides and PCR detected by SYBR green were performed in the same plate as controls. After the 8 cycles, 2µL first PCR products were added to each of 2 wells, mixed with 8µl PCR reaction mix [5µl of 2x Lightcycler Probe Master Mix, 0.2μM final concentration of allele specific primer set *IFNB1* MF/R, and 0.1μM final concentration of Roche LNA probe #79]. The second PCR amplification was carried out using a polymerase activation at 95°C for 10 minutes, followed for 50 cycles at 94ºC for 10s, 62ºC for 20s and 72ºC for 10s. The PCR amplification curves (Supplementary **Figure S5A**) and regression plot (inset in Supplementary **Figure S5A**) of the real-time hemi-nested PCR for the *IFNB1* gene demonstrated that the quantity of *IFNB1* can be detected accurately down to 4 copies of template, where the standard error in cycle numbers (Cp) expands only to 1.3 for 12 repeated measurements. Furthermore, the consistency or variation of PCR efficiency among the wells in the PCR plate was tested by running a parallel real-time PCR with SYBR green readout and the primer *IFNB1* OF that only uses the control oligonucleotides, present at 200 copies, as its template (Supplementary **Figure S5B**). This control assay demonstrated a very tight distribution in the presence of the genomic standard dilution series, suggesting PCR efficiency was independent of genomic DNA copy number. Finally, we demonstrated that omission of the control oligonucleotides had no effect on the overall accuracy of the hemi-nested TaqMan PCR using LNA probes (data not shown).

To evaluate the multiplex hemi-nested PCR of *IFNB1* and *DDX58*, total RNAs were extracted from DCs from the same donor studied in for the single cell RT described below and were diluted to approximately 1 copy as determined by the PCR result above for genomic DNA. The 2-step PCR protocol was carried out as described above except that the first PCR used 5µL of 2xAccuRT PCR reaction mix [2xAccuRT buffer, 4mM magnesium acetate, 0.2μM universal primer sets for each *IFNB1* and *DDX58*, 200copies/well *IFNB1* and *DDX58* control oligonucleotides, 0.2mM each dNTP (dUTP) and 0.375U/µL AccuRT with aptamer (a dual functional RNA-DNA polymerase kindly provided by Dr. Tom Myers of Roche Molecular Systems)]. The first RT-PCR was carried out with a 65°C reverse transcription for 30 min and 8 cycles of 94°C for 15s and 60°C for 50s. After the 8 cycles, 2µL of first PCR products were added to 4 wells, mixed with 8μL PCR reaction mix described above for genomic DNA, containing either 0.2µM *IFNB1* or *DDX58* allele specific primer set and the corresponded Roche LNA probe (#79 for *IFNB1* and #6 for *DDX58*). Multiplex RT-PCR measurement of *IFNB1* and *DDX58* transcripts (Supplementary **Figure S6**) were performed on serially diluted total RNA samples. We evaluated 4 10-fold dilutions for *IFNB1* (Supplementary **Figure S6A**)*,* whereas we evaluated only 3 dilutions for *DDX58* (Supplementary **Figure S6B**) because the lower expression level of *DDX58* led to less than 1 copy of template at the lowest dilution. The measurement of both *DDX58* and *IFNB1* transcripts by this hemi-nested PCR method showed similar precision when compared to the single *IFNB1* gene using the genomic DNA standards (Supplementary **Figure S5A**).

## Table of PCR primers and oligonucleotides for the real time PCR

| PCR primers for human total RNA real time RT-PCR assays | |
| --- | --- |
| IFNB1 R  IFNB1 F  DDX58 R  DDX58 F  ACTB R  ACTB F | TTCTTCCAGGACTGTCTTCAGATGGTT  CACTGGCTGGAATGAGACTATT  GAAGCACTTGCTACCTCTTGC  ATGTGGGCAATGTCATCAAA  GTGGACTTGGGAGAGGACTG  ACTGGAACGGTGAAGGTGAC |
| PCR primers and internal control oligonucleotides for single cell real time RT-PCR assays of human *IFNB1* and *DDX58** | |
| *IFNB1* MF**  *IFNB1* OF****  *IFNB1* oligo-  nucleotide  *DDX58* MF**  *DDX58* OF****  *DDX58* oligo-  nucleotide | ATCCCGTTGATCTCCAGGGAA  ATCCCGTTGATCTCCAGGGTT  AAAGATTCATCTAGCACTGGCTGGAATGAGACTATTGTTGAG***TT*** **CCTCCT**GGCTAATGTCTATCATCAGATAAACCTCTGAAGACAGT CCTGGAAGAAA  ATGTGGGCAATGTCATCAAAATGATCCAA  ATGTGGGCAATGTCATCAAAATGATCCTT  GAGTATGTGGGCAATGTCATCAAAATGATCC***TT***ACCAG**AGGCAG** AGGAAGAGCAAGAGGTAGCAAGTGCTTCCTTCTGAC |

***** Internal control oligonucleotides for *IFNB1* and *DDX58* were designed to have the identical cDNA sequence but with a 2nt substitution (letter bold italicized) and lack the 6nt Roche probe binding site (sequences bold underlined)

** Both MF primers were designed to have 2nt at 3’ end which specifically matched with the cDNA template, while OF primers were designed to specifically anneal with the PCR products initiated from the control oligonucleotides.
