# Supplementary material for: Role of Cell-to-Cell Variability in Activating a Positive Feedback Antiviral Response in Human Dendritic Cells
Source: PLoS One. 2011 Feb 8;6(2):e16614. doi: 10.1371/journal.pone.0016614 (PMC3035661; doi:10.1371/journal.pone.0016614)
Supplement: Text S2 — Changing the Variance and Maintaining Early Responder Percentage. (DOCX) [file pone.0016614.s009.docx]

## Supplementary Text S2: Changing the Variance and Maintaining Early Responder Percentage

The initial *DDX58* concentration is distributed according to a log-normal distribution. The activation of *IFNB1* in infected cells depends on the concentration of *DDX58* according to a Michaelis-Menten term $K_{IFN}\frac{\left[\gamma\cdot RIG\right]^{H_{IFN}}}{1+\left[\gamma\cdot RIG\right]^{H_{IFN}}}$. The parameter $1/\gamma$ determines the critical concentration of *DDX58* that activates the cell. When reducing the variance of the log normal distribution, we also decrease the value of $1/\gamma$ so that the probability of any cell having a concentration of *DDX58* larger than $1/\gamma$ remains constant, thus roughly maintaining the same percentage of early responder cells. A log-normal distribution is defined by $f(x;\mu,\sigma)=\frac{1}{x\sigma\sqrt{2\pi}}e^{-\frac{\left(\ln x-\mu\right)^{2}}{2\sigma^{2}}}$, where $\mu$ and $\sigma^{2}$ are the mean and variance of $\ln x$, and its cumulative distribution function is $F(x;\mu,\sigma)=\frac{1}{2}+\frac{1}{2}\text{erfc}\left(-\frac{\ln x-\mu}{\sigma\sqrt{2}}\right)$, where $\text{erfc}$ is the complementary error function. Denoting the mean and variance of the log-normal distribution by $\mu_{L}$ and $\sigma_{L}^{2}$ respectively we get and $\sigma^{2}=\ln\left(1+\frac{\sigma_{L}^{2}}{\mu_{L}^{2}}\right)$. We want to change $\sigma_{L}^{2}$ while keeping both $\mu_{L}$ and $F(1/\gamma;\mu,\sigma)$constant, which translates to keeping $\frac{\ln\gamma+\mu}{\sigma\sqrt{2}}=C$. Replacing $\mu$ and $\sigma$ above we get $\ln\gamma=C\sqrt{2\ln\left(1+\frac{\sigma_{L}^{2}}{\mu_{L}^{2}}\right)}-\ln\mu_{L}-\frac{1}{2}\ln\left(1+\frac{\sigma_{L}^{2}}{\mu_{L}^{2}}\right)$. The constant *C* can be determined by the original values of $\mu_{L}$, $\gamma$ and $\sigma_{L}^{2}$.
